# Problem of Existence of Joint Distribution on Quantum Logic †

**DOI:** 10.3390/e26121121

**Published:** 2024-12-21

**Authors:** Oľga Nánásiová, Karla Čipková, Michal Zákopčan

**Affiliations:** Faculty of Electrical Engineering and Information Technology, Slovak University of Technology in Bratislava, Ilkovičova 3, 841 04 Bratislava, Slovakia; karla.cipkova@stuba.sk (K.Č.); michal.zakopcan@stuba.sk (M.Z.)

**Keywords:** generalized probability, probability space, marginal distribution, joint distribution, quantum logic, non-compatibility, maps for simultaneous measurements

## Abstract

This paper deals with the topics of modeling joint distributions on a generalized probability space. An algebraic structure known as quantum logic is taken as the basic model. There is a brief summary of some earlier published findings concerning a function *s*-map, which is a mathematical tool suitable for constructing virtual joint probabilities of even non-compatible propositions. The paper completes conclusions published in 2020 and extends the results for three or more random variables if the marginal distributions are known. The existence of a (n+1)-variate joint distribution is shown in special cases when the quantum logic consists of at most *n* blocks of Boolean algebras.

## 1. Introduction

One of the goals of modeling on orthomodular lattices, especially on quantum logics, is to study how to simulate probability so that it preserves the properties of the normed measure known from Boolean algebras. The problem was originally formulated in [1]. Later, it was discussed in many publications from a mathematical (e.g., [2,3,4,5,6,7,8]), physical (e.g., [9,10,11,12,13,14,15]), or philosophical/economical/social science point of view (e.g., [16,17,18,19]). One can find a wide overview of works within this topic up to the year 1992 in [20].

To measure probability for logical connectives of propositions in Boolean algebras, one can use the classical probability measure, also known as *state*. However, such modeling in orthomodular lattices, which are non-distributive generalizations of Boolean algebras, leads to difficulties (see, e.g., [21]). These difficulties are associated with a need to measure probabilities for logical connectives of propositions, which are not compatible. Quantum logic allows for the modeling of situations with non-compatible events, i.e., events that are not simultaneously measurable/observable. Methods of quantum logic appear in many domains of application, such as in economic models, data processing, and quantum mechanics (see, e.g., [1,13,14,15,18,22,23,24,25,26,27]). There is not enough space here to list all of the publications devoted to the wide scope of quantum logic applications.

Building a generalized probability theory requires introducing the concept of conditional probability (see, e.g., [28,29,30]) and defining the notion of a joint distribution (see, e.g., [31,32,33,34,35]) having the same properties as these notions have in classical probability space. In order to build such a theory including incompatible random variables, there is a need to introduce the concept of conditional probability for non-compatible random events and to follow the mentioned properties of notions.

The calculus for non-compatible observables can be found in [36]. In our paper, we follow the definition of conditional state (conditional probability) introduced in [37] and especially the function named *s*-map [38] derived from it. The notation “*s*” in *s*-map signifies a map for *simultaneous* measurements. This notion was studied in numerous papers (for all see e.g., [39,40]) and we find it a very useful mathematical tool suitable for constructing virtual joint probabilities of a pair of non-compatible propositions in quantum logic. Using an *s*-map *p*, the probability of the conjunction of propositions is modeled as a value that *p* takes on a pair of these propositions. Afterward, the value p(a,b), with *a* and *b* non-compatible propositions, could be understood as the truth-value of the ‘counterfactual conjunction’ of propositions, i.e., ‘what would the probability of simultaneous verification of propositions *a* and *b* be if we were able to perform it’. In general, the value of p(a,b) depends on the order of non-compatible propositions, i.e., if *p* is not a commutative *s*-map there are non-compatible propositions for which p(a,b)≠p(b,a).

The present paper deals with the topics of *n*-variable additive mappings on quantum logic. There is a straightforward continuity to [39,41], and it is an extension of results published in [42]. The topic itself was discussed with Prof. Jaroslaw Pykacz (deceased in 2021) over his last stay at the Slovak University of Technology in Bratislava.

This paper deals with the existence of joint distribution for three or more random variables in case we know their marginal distributions (similar problems were studied, e.g., in [43,44,45,46]). The paper completes the conclusions of [42] for three random variables in a special case when a quantum logic consists of two blocks of Boolean algebras. Analogous reasoning is then extended to n+1 random variables for a quantum logic consisting of at most *n* blocks of Boolean algebras.

The paper is organized as follows: Section 2 recalls some basic notions and their properties. In Section 3, one can find the use of the *s*-map properties in an example of trivariate *s*-map and its bivariate marginal maps. Section 4 is devoted to the derivation of conditions for the existence of trivariate extension of bivariate *s*-maps, (n+1)-variate extension of *n*-variate *s*-maps, respectively.

## 2. Preliminaries

In this section, we recall fundamental notions of orthomodular lattice, orthogonality, compatibility, probability measure, quantum logic, *s*-maps, observables and joint distributions, *n*-variate *s*-maps, and their properties. For more details see [3,5,37,38,39,40,47,48].

### 2.1. Quantum Logic

**Definition 1.**  *Let L be a nonempty set endowed with a partial ordering ≤. Let there exist the greatest element ($1_{L}$) and the smallest element ($0_{L}$). Let there be defined the (lattice) operations supreme (∨), infimum (∧) and a map ′:L→L with the following properties:*

*(i)*  *${\left(a^{\prime }\right)}^{\prime }=a$ (in what follows instead of ${\left(a^{\prime }\right)}^{\prime }$ we write $a^{\prime \prime }$);*
*(ii)*  *if a≤b then $b^{\prime }\leq a^{\prime }$;*
*(iii)*  *$a\vee a^{\prime }=1_{L}$;*
*(iv)*  *if a≤b then $b=a\vee (a^{\prime }\wedge b)$ (orthomodular law).*

*Then $\left(L,0_{L},1_{L},\vee ,\wedge {,}^{\prime }\right)$ is called the* orthomodular lattice *(briefly OML).*

**Definition 2.**  *Two elements a,b from OML L are called:*

*(i)*  orthogonal *if and only if $a\leq b^{\prime }$ (in what follows we write a⊥b);*
*(ii)*  compatible *(we write a↔b) if and only if there exist pairwise orthogonal elements $a_{1},b_{1},c\in L$ with*
  $$a=a_{1}\vee c\;and\;b=b_{1}\vee c.$$

The elements $a_{1},b_{1},c\in L$ are unambiguously determined and satisfy [3]

$$a_{1}=a\wedge b^{\prime },\;b_{1}=a^{\prime }\wedge b,\;c=a\wedge b.$$

Instead of the previous definition (Definition 2) of compatibility of the elements from *L*, it is common to use a shorter version of it, which says the elements a,b∈L are compatible if $a=\left(a\wedge b\right)\vee \left(a\wedge b^{\prime }\right)$.

If a∈L is compatible to b∈L, then *b* is also compatible to *a*. Moreover, a↔a, $a↔a^{\prime }$, $a↔1_{L}$ and $a↔0_{L}$. If a≤b then a↔b and if c⊥d, then c↔d. The compatibility relation does not have a transitive property, i.e., if a↔b and b↔c, no one can automatically conclude that a↔c.

If *L* is a Boolean algebra, then two arbitrary elements from *L* are compatible. Furthermore each OML is a union of Boolean subalgebras with non-empty intersections while the maximal Boolean subalgebra is referred to as a block. For any element a∈L there exists at least one block containing *a*.

**Definition 3.**  *A* probability measure *(alternatively called a* state *on L) is a mapping m:L→[0,1] satisfying*

*(i)*  *$m(1_{L})=1$;*
*(ii)*  *if a⊥b then m(a∨b)=m(a)+m(b).*

There are three types of OMLs according to the number of states: OMLs with no state, OMLs with exactly one state, and OMLs with an uncountable number of states (see, e.g., [49]). An OML, which is of the last type, is usually called a *quantum logic* (abbr. QL). From the algebraic point of view, a QL *L* is an orthomodular lattice such that for every orthogonal element a,b∈L, we have a∨b∈L and a∧b∈L.

Not every OML can be used for modeling uncertainty, or for modeling generalized probability theory. We consider a QL as a type of OML suitable for such modeling. Throughout present paper *L* is assumed to be a quantum logic.

### 2.2. S-Maps

Among several possible approaches to the problem of joint distribution (a measure of virtual conjunction), there is one applying the notion of s-map, introduced by Nánásiová in [38].

**Definition 4.**  *A map p:L×L→[0,1] is called a* map for simultaneous measurements *(abbr.* s-map*) if the following conditions are satisfied:*

*(s1)*  *$p(1_{L},1_{L})=1$;*
*(s2)*  *if a⊥b then p(a,b)=0;*
*(s3)*  *if a⊥b then for any c∈L:*
  p(a∨b,c)=p(a,c)+p(b,c),
  p(c,a∨b)=p(c,a)+p(c,b).

This definition is the immediate consequence of the definition of conditional state on quantum logic (see in [37]).

Here we mention three properties of *s*-maps proved in [38] which will play an important role in our further considerations:

(N1) The map $m_{p}:L\to \left[0,1\right]$ with $m_{p}\left(a\right)=p\left(a,a\right)=p\left(1_{L},a\right)=p\left(a,1_{L}\right)$ is a state on *L*. Such a state is referred to as a *state generated by p*.
(N2) If a↔b then $p\left(a,b\right)=m_{p}\left(a\wedge b\right)=p\left(b,a\right)$.
(N3) $p\left(a^{\prime },b^{\prime }\right)=1-m_{p}\left(a\right)-m_{p}\left(b\right)+p\left(a,b\right)$ for arbitrary elements a,b∈L.

The property (N2) shows that the value p(a,b) of compatible propositions (a↔b) corresponds to the value that a state $m_{p}$ generated by *p* assigns to the meet a∧b, representing a conjunction of *a* and *b*. On the other hand, *s*-maps can be seen as providing probabilities of ‘virtual’ conjunctions of propositions, even non-compatible ones.

There exist OMLs admitting no states as Greechie showed in [21]. Of course, no *s*-map can be defined on such OMLs. On the contrary, there exist a lot of *s*-maps on OMLs with unital sets of states (Propositions 1.1 and 2.2 of [38]).

### 2.3. *n*-Variate S-Maps and Their Properties

At first we remind the crucial definition of an *n*-variate *s*-map (see in [40]).

**Definition 5**  ([40])**.** *Let L be an OML. The map $p^{n}:L^{n}\to \left[0,1\right]$ conforming to the conditions:*

*(s1’)*  *$p^{n}\left(1_{L},\ldots ,1_{L}\right)=1$;*
*(s2’)*   *if $a_{i}⊥a_{j}$ for some i,j∈{1,⋯,n}, then $p^{n}\left(a_{1},\ldots ,a_{n}\right)=0$;*
*(s3’)*  *if $b_{i}⊥c_{i}$ for some i∈{1,⋯,n}, then*
  $$\;p^{n}\left(a_{1},\ldots ,b_{i}\vee c_{i},\ldots ,a_{n}\right)=p^{n}\left(a_{1},\ldots ,b_{i},\ldots ,a_{n}\right)+p^{n}\left(a_{1},\ldots ,c_{i},\ldots ,a_{n}\right);$$

*is referred to as an n-variate s-map on L.*

Let us remark that the s-map defined in the previous text is a bivariate s-map in the definition just mentioned (n=2).

**Remark 1**  ([40])**.** *Let L be a Boolean algebra and $m_{p}:L\to \left[0,1\right]$ be mapping defined by $m_{p}\left(a\right)=p^{n}\left(a,\cdots ,a\right)$, then $m_{p}$ is an additive measure induced by the s-map $p^{n}$ satisfying*

$$p^{n}\left(a_{1},a_{2},\ldots ,a_{n}\right)=m_{p}\left(a_{1}\wedge a_{2}\wedge \ldots \wedge a_{n}\right).$$

In general, an *s*-map is not invariant with respect to permutations of elements, therefore

$$p^{n}\left(a_{1},a_{2},\ldots ,a_{n}\right)=p^{n}\left(\pi \left(a_{1},a_{2},\ldots ,a_{n}\right)\right),$$

where $\pi (a_{1},\ldots ,a_{n})$ denotes a permutation of $\left(a_{1},\ldots ,a_{n}\right)$, does not hold in general. We recommend the reader to see [40].

In case $p^{n}\left(a_{1},a_{2},\ldots ,a_{n}\right)\neq p^{n}\left(\pi \left(a_{1},a_{2},\ldots ,a_{n}\right)\right)$, mapping $p^{n}$ may be referred to as a contextual s-map since the probability value for elements $a_{1},a_{2},\ldots ,a_{n}$ depends on the order of input variables.

**Proposition 1**  ([40])**.** *Let $p^{n}$ be an n-variate s-map on a QL L and let $\left(a_{1},\ldots ,a_{n}\right)\in L^{n}$. Then*

*(a)*  *a map $m_{p}:L\to \left[0,1\right]$ satisfying $m_{p}\left(a\right):=p^{n}\left(a,\ldots ,a\right)$, a∈L, is a state on L, it means*
  $$p^{n}\left(a,\ldots ,a\right)=p^{n}\left(\pi \left(a,\ldots ,1_{L}\right)\right)=p^{n}\left(\pi \left(a,\ldots ,1_{L},1_{L}\right)\right)=p^{n}\left(\pi \left(a,1_{L},\ldots ,1_{L}\right)\right),$$
  *where π is any permutation;*
*(b)*  *for any $\left(a_{1},\ldots ,a_{n}\right)\in L^{n}$$p^{n}\left(a_{1},\ldots ,a_{n}\right)\leq m_{p}\left(a_{i}\right)$ for each i∈{1,2,⋯,n};*
*(c)*  *if $a_{i}↔a_{j}$ for some i≠j, i,j∈{1,⋯,n} then*
  $$\begin{array}{ccc} p^{n}\left(a_{1},\ldots ,a_{n}\right) & = & p^{n}\left(a_{1},\ldots ,a_{i-1},a_{i}\wedge a_{j},a_{i+1},\ldots ,a_{j-1},a_{j}\wedge a_{i},a_{j+1},\ldots ,a_{n}\right)\\ & = & p^{n}\left(\pi \left(a_{1},\ldots ,a_{n}\right)\right) \end{array}$$
  *for arbitrary permutation π,*
*(d)*  *if $a_{i}=1_{L}$ for some i∈{1,⋯,n} then*
  $$p^{n}\left(a_{1},\ldots ,a_{n}\right)=p^{n}\left(a_{1},\ldots ,a_{i-1},a_{j},a_{i+1},\ldots ,a_{n}\right)$$
  *for each j∈{1,⋯,n}.*

**Definition 6**  ([40])**.** *Let $p^{n}:L^{n}\to \left[0,\;1\right]$ be an n-variate s-map. Let k<n, k∈N. The map $p^{k}:L^{k}\to \left[0,1\right]$ satisfying*

$$p^{k}\left(a_{1},a_{2},\ldots ,a_{k}\right)=p^{n}\left(a_{1},a_{2},\ldots ,a_{k},1_{L},\ldots ,1_{L}\right)$$

*for any $a_{i}\in L$, i=1,2,⋯,k is referred to as a marginal s-map, and the original n-variate s-map $p^{n}$ is called an extension of $p^{k}$.*

The fact that $1_{L}↔a$ for any a∈L and the assertion of Proposition 1 imply that each marginal *s*-map is invariant with respect to (abbreviation w.r.t.) permutations. In the sequel, we will study only n-variate *s*-maps which are invariant w.r.t. permutations, i.e.,

$$p^{n}\left(a_{1},\ldots ,a_{n}\right)=p^{n}\left(\pi \left(a_{1},\ldots ,a_{n}\right)\right)$$

for any permutation π and any $\left(a_{1},\ldots ,a_{n}\right)\in L^{n}$. Bivariate s-map invariant w.r.t. permutations will be called *commutative*.

**Remark 2.**  *If a↔b and c∈L then*

$$\begin{array}{cc} p^{3}\left(a,b,c\right) & =p^{3}\left(\left(a\wedge b\right)\vee \left(a\wedge b^{\prime }\right),b,c\right)=p^{3}\left(\left(a\wedge b\right),b,c\right)+\underset{0}{\underbrace{p^{3}\left(\left(a\wedge b^{\prime }\right),b,c\right)}}=\\ \; & =p^{3}\left(a\wedge b,b,c\right)=p^{3}\left(a\wedge b,b\wedge a,c\right)=p^{3}\left(a\wedge b,1_{L},c\right)=\\ \; & =p^{3}\left(a\wedge b,c\vee c^{\prime },c\right)=p^{3}\left(a\wedge b,c,c\right)+\underset{0}{\underbrace{p^{3}\left(a\wedge b,c^{\prime },c\right)}}=\\ \; & =p^{3}\left(\pi \left(a\wedge b,1_{L},c\right)\right)=p^{3}\left(\pi \left(a\wedge b,c,c\right)\right). \end{array}$$

*That means $p^{3}\left(a\wedge b,x,c\right)$ is invariant w. r. t. order of input variables for any $x\in \{a\wedge b,a,b,c,1_{L}\}$.*

### 2.4. Observables and Joint Distributions

From a mathematical point of view, the definition of the concept of an observable depends on the basic structure that one uses for modeling uncertainty.

We will call an *observable* a homomorphism *x* from Borel subsets of real line B(R) to a quantum logic *L* such that x(∅)=0, x(R)=1, $x\left(E^{c}\right)={\left(x\left(E\right)\right)}^{\prime }$ and x(E∪F)=x(E)∨x(F) for any E,F∈B(R) (see e.g., [3,50]). We denote R(x) the *range* of observable *x*, namely R(x)={x(E):E∈B(R)}.

The observables *x*, *y* are called compatible (x↔y) or simultaneously measurable, if for arbitrary E,F∈B(R) we have x(E)↔y(F). The observables *x*, *y* are compatible if and only if a↔b for any a∈R(x) and any b∈R(y). In the case where we consider two simultaneously measurable observables, all propositions from the ranges of observables could be then interpreted in the way of classical (Boolean) logic.

The problem of introducing some calculus for non-compatible observables has been studied since Birkhoff and von Neumann [1].

In order to introduce a similar calculus for non-compatible observables as in the classical theory of probability, a concept similar to a joint distribution is needed. To meet several approaches to this problem we recommend the reader to see, e.g., [3,5,40,50,51,52,53,54,55,56,57,58]. In order to define the notion of a joint distribution, we will follow the concept of an s-map.

Let *x*, *y* be observables on *L* and *p* be an s-map. Then the function

$$F_{x,y}\left(t,s\right)=p\left(x\left(-\infty ,t\right),y\left(-\infty ,s\right)\right)$$

is the joint distribution for observables x,y (e.g., [36]). If x↔y, then

$$F_{x,y}\left(t,s\right)=p\left(x\left(-\infty ,t\right),y\left(-\infty ,s\right)\right)=m_{p}\left(x\left(-\infty ,t\right)\wedge y\left(-\infty ,s\right)\right)$$

for any t,s∈R. Since $m_{p}$ is a probability measure, the function $F_{x,y}\left(\cdot ,\cdot \right)$ is the joint distribution for random variables generated with observables *x* and *y*.

The definition of a joint distribution could be extended for three (or more) observables *x*, *y*, *z* in a natural manner:

$$F_{x,y,z}\left(t,s,u\right)=p\left(x\left(-\infty ,t\right),y\left(-\infty ,s\right),z\left(-\infty ,u\right)\right).$$

## 3. Bivariate s-Map Extension

Following the conditions from Definition 6 if *p* is a bivariate commutative *s*-map on *L* then the trivariate extension of *p* is an *s*-map $p^{3}:L^{3}\to \left[0,1\right]$ satisfying

$$p\left(a,b\right)=p^{3}\left(a,b,1_{L}\right).$$

There is a straightforward way to find a function that satisfies this condition. One can choose, e.g., $p^{3}\left(a,b,c\right)=\min\left\{p\left(a,b\right),p\left(a,c\right),p\left(b,c\right)\right\}$. However, such a function would not necessarily be an *s*-map because of violating some conditions following from Definition 5. The following example illustrates such a situation.

**Example 1.**  *Let L be a horizontal sum of three 4-element Boolean algebras $\left\{0_{L},u,u^{\prime },1_{L}\right\}$, where u∈{a,b,c}. Consider a bivariate commutative s-map p. Since*

$$p\left(u,u\right)=p\left(u,1_{L}\right)=p\left(u,v\vee v^{\prime }\right)=p\left(u,v\right)+p\left(u,v^{\prime }\right)$$

*for some $v\in \{a,b,c,1_{L}\}$, Table 1 defines the following s-map:*

*We show it is impossible to extend the bivariate map p to a trivariate s-map $p^{3}$. To construct such map, one has to choose $p^{3}\left(x,y,z\right)$ in such way that*

$$0\leq p^{3}\left(x,y,z\right)\leq \min\left\{p\left(x,y\right),p\left(x,z\right),p\left(y,z\right)\right\}$$

*for any mutually distinct $x,y,z\in \{a,a^{\prime },b,b^{\prime },c,c^{\prime }\}$. In addition, the sum of all $p^{3}\left(x,y,z\right)$ chosen this way has to be equal to 1.*

*Denote:*

$$\begin{array}{cc} Q_{1} & =\min\{p(a,b),p(a,c),p(b,c)\}=\min\{0.1,0.1,0\}=0,\\ Q_{2} & =\min\left\{p\left(a,b\right),p\left(a,c^{\prime }\right),p\left(b,c^{\prime }\right)\right\}=\min\left\{0.1,0,0.2\right\}=0,\\ Q_{3} & =\min\left\{p\left(a,b^{\prime }\right),p\left(a,c\right),p\left(b^{\prime },c\right)\right\}=\min\left\{0,0.1,0.4\right\}=0,\\ Q_{4} & =\min\left\{p\left(a,b^{\prime }\right),p\left(a,c^{\prime }\right),p\left(b^{\prime },c^{\prime }\right)\right\}=\min\left\{0,0,0.4\right\}=0,\\ Q_{5} & =\min\left\{p\left(a^{\prime },b\right),p\left(a^{\prime },c\right),p\left(b,c\right)\right\}=\min\left\{0.1,0.3,0\right\}=0,\\ Q_{6} & =\min\left\{p\left(a^{\prime },b\right),p\left(a^{\prime },c^{\prime }\right),p\left(b,c^{\prime }\right)\right\}=\min\left\{0.1,0.6,0.2\right\}=0.1,\\ Q_{7} & =\min\left\{p\left(a^{\prime },b^{\prime }\right),p\left(a^{\prime },c\right),p\left(b^{\prime },c\right)\right\}=\min\left\{0.8,0.3,0.4\right\}=0.3,\\ Q_{8} & =\min\left\{p\left(a^{\prime },b^{\prime }\right),p\left(a^{\prime },c^{\prime }\right),p\left(b^{\prime },c^{\prime }\right)\right\}=\min\left\{0.8,0.6,0.4\right\}=0.4, \end{array}$$

*then*

$$\sum_{i=1}^{8}Q_{i}=0.8<1.$$

*We can conclude there is no trivariate extension $p^{3}$ of the bivariate s-map p.*

**Remark 3.**  *Let $p:L^{2}\to \left[0,\;1\right]$ be a bivariate s-map on L. Then the function $d_{p}:L^{2}\to \left[0,\;1\right]$ such that*

$$d_{p}\left(a,b\right)=p\left(a^{\prime },b\right)+p\left(a,b^{\prime }\right)=m_{p}\left(a\right)+m_{p}\left(b\right)-2p\left(a,b\right)$$

*can be interpreted as a measure of distance for a,b∈L. This function has been studied, e.g., in [39,41,47].*

*Generally there exist a,b∈L and an s-map p such that $d_{p}\left(a,b\right)\neq d_{p}\left(b,a\right)$. Moreover, there is no guarantee for this mapping to be a pseudo-metric, i.e., the triangle inequality*

(1) $$\begin{array}{c} d_{p}\left(a,b\right)\leq d_{p}\left(a,c\right)+d_{p}\left(c,b\right) \end{array}$$

*can be infringed.*

*Under the assumption of B being a Boolean algebra, a mapping $d_{p}$ is pseudo-metric, namely for arbitrary a,b,c∈B the triangle inequality *(1)* is fulfilled. Furthermore, $d_{p}\left(a,b\right)=d_{p}\left(b,a\right).$*

**Proposition 2**  ([39], Proposition 3)**.** *Let L be a quantum logic in which*

$$L=\{a_{1},a_{1}^{\prime },a_{2},a_{2}^{\prime },a_{3},a_{3}^{\prime },0_{L},1_{L}\}$$

*is a horizontal sum of three 4-element Boolean algebras $\left\{a_{i},a_{i}^{\prime },0_{L},1_{L}\right\}$, i=1,2,3. Let p be a commutative bivariate s-map on L and let $d_{p}$ be the d-map induced by p. The s-map p is a marginal one if and only if $d_{p}$ satisfies the triangle inequality on L.*

**Proposition 3**  ([42], Proposition 3)**.** *Let p be a commutative bivariate s-map on a quantum logic L. Then a trivariate s-map, which is an extension of p exists if and only if $d_{p}$ satisfies the triangle inequality on L.*

## 4. Existence of Trivariate Extensions of Bivariate *s*-Maps

This section is devoted to the study of conditions for the existence of extensions of a bivariate s-map *p* to trivariate s-maps $p^{3}$. Proposition 4 extends the conclusions of Proposition 3 and Proposition 2, respectively.

**Proposition 4.**  *Let a QL L consist of two blocks $B_{1},B_{2}$ ($L=B_{1}\cup B_{2}$ and $\left\{0_{L},1_{L}\right\}\subseteq B_{1}\cap B_{2}$), where $B_{i}$ is a Boolean algebra, i=1,2. Then any commutative bivariate s-map p can be extended to a trivariate s-map $p^{3}$.*

**Proof of Proposition 4.**  Let $L=B_{1}\cup B_{2}$, where $B_{1},B_{2}$ are Boolean algebras and $\left\{0_{L},1_{L}\right\}\subseteq B_{1}\cap B_{2}$. By means of *p* (a given bivariate commutative s-map), we intend to define a mapping $g:L^{3}\to \left[0,1\right]$. As there are two blocks and three variables of *g*, we expect it to be invariant with respect to any permutation π and any triple $\left(x_{1},x_{2},x_{3}\right)\in L^{3}$, i.e.,

$$g\left(\pi \left(x_{1},x_{2},x_{3}\right)\right)=g\left(x_{1},x_{2},x_{3}\right)$$

since at least two of $x_{1},x_{2},x_{3}$ are compatible (see Proposition 1c).

Furthermore, we expect *g* to be an extension of $p:L^{2}\to \left[0,1\right]$, therefore (without loss of generality let $x_{1}↔x_{2}$) we have

$$g\left(x_{1},x_{2},x_{3}\right)=g\left(x_{1}\wedge x_{2},x_{1}\wedge x_{2},x_{3}\right)=g\left(1_{L},x_{1}\wedge x_{2},x_{3}\right).$$

In this manner, we naturally come to a unique way how to define $g:L^{3}\to \left[0,1\right]$:

$$g\left(x_{1},x_{2},x_{3}\right)=p\left(x_{i}\wedge x_{j},\;x_{k}\right)$$

where i,j,k∈{1,2,3},i,j,k are pairwise distinct and $x_{i}↔x_{j}$.

The correctness of the definition of mapping *g* follows directly from Proposition 1c and Remark 2.

It is easy to see that mapping *g* defined in this way is permutation-free, which means that $g\left(\pi \left(x_{1},\;x_{2},\;x_{3}\right)\right)=g\left(x_{1},\;x_{2},\;x_{3}\right)$ for all $\left(x_{1},\;x_{2},\;x_{3}\right)\in L^{3}$ and for arbitrary permutation π of elements $x_{1},\;x_{2},\;x_{3}\in L$.

In addition, mapping *g* is really an extension of *p* since:

$$g\left(1_{L},\;x_{2},\;x_{3}\right)=p\left(1_{L}\wedge x_{2},\;x_{3}\right)=p\left(x_{2},\;x_{3}\right).$$

Having *g* defined, let us verify conditions (s1’) – (s3’) of Definition 5. To further simplify our reasoning we can, without loss of generality, suppose that

$x_{1}\in B_{1},\;x_{1}↔x_{2}$:

(s1’) $g\left(1_{L},\;1_{L},\;1_{L}\right)=p\left(1_{L}\wedge 1_{L},\;1_{L}\right)=1,$
(s2.1’) if $x_{1}⊥x_{2}$, then
  $$g\left(x_{1},\;x_{2},\;x_{3}\right)=p\left(x_{1}\wedge x_{2},\;x_{3}\right)=p\left(0_{L},\;x_{3}\right)=0,$$
(s2.2’) if $x_{1}⊥x_{3}$, then $x_{1}\wedge x_{2}⊥x_{3}$ and thus
  $$g\left(x_{1},\;x_{2},\;x_{3}\right)=p\left(x_{1}\wedge x_{2},\;x_{3}\right)=0,$$
(s2.3’) if $x_{2}⊥x_{3}$, then likewise the previous case $x_{1}\wedge x_{2}⊥x_{3}$ and
  $$g\left(x_{1},\;x_{2},\;x_{3}\right)=p\left(x_{1}\wedge x_{2},\;x_{3}\right)=0,$$
(s3’) It remains to show additivity of *g* in each coordinate. Let ${\dot{x}}_{1},\;{\ddot{x}}_{1}\in L$ be such that $x_{1}={\dot{x}}_{1}\vee {\ddot{x}}_{1},\;{\dot{x}}_{1}⊥{\ddot{x}}_{1}$. It is clear that both ${\dot{x}}_{1}$ and ${\ddot{x}}_{1}$ are compatible with $x_{1}$. Thus ${\dot{x}}_{1},\;{\ddot{x}}_{1}\in B_{1}$ as well.
  First, let us compute $g\left(x_{1},\;x_{2},\;x_{3}\right)=g\left({\dot{x}}_{1}\vee {\ddot{x}}_{1},\;x_{2},\;x_{3}\right)$.
  If $x_{2}↔x_{3}$, we obtain
  $$\begin{array}{ccc} g({\dot{x}}_{1}\vee {\ddot{x}}_{1},\;x_{2},\;x_{3}) & = & p(x_{2}\wedge x_{3},\;{\dot{x}}_{1}\vee {\ddot{x}}_{1})=\\ & = & p\left(x_{2}\wedge x_{3},\;{\dot{x}}_{1}\right)+p\left(x_{2}\wedge x_{3},\;{\ddot{x}}_{1}\right)=\\ & = & g\left({\dot{x}}_{1},\;x_{2},\;x_{3}\right)+g\left({\ddot{x}}_{1},\;x_{2},\;x_{3}\right). \end{array}$$
  If $x_{2}↮x_{3}$, then $x_{1}$ is compatible with at least one of $x_{2},\;x_{3}$. Without loss of generality let $x_{1}↔x_{2}$. Furthermore, since ${\dot{x}}_{1},\;{\ddot{x}}_{1},\;x_{2}\in B_{1}$, we obtain
  $$\begin{array}{ccc} g({\dot{x}}_{1}\vee {\ddot{x}}_{1},\;x_{2},\;x_{3}) & = & p(\left({\dot{x}}_{1}\vee {\ddot{x}}_{1}\right)\wedge x_{2},\;x_{3})=\\ & = & p(\left({\dot{x}}_{1}\wedge x_{2}\right)\vee \left({\ddot{x}}_{1}\wedge x_{2}\right),\;x_{3}). \end{array}$$
  Realizing that $\left({\dot{x}}_{1}\wedge x_{2}\right)⊥\left({\ddot{x}}_{1}\wedge x_{2}\right)$ we conclude
  $$\begin{array}{ccc} g({\dot{x}}_{1}\vee {\ddot{x}}_{1},\;x_{2},\;x_{3}) & = & p\left({\dot{x}}_{1}\wedge x_{2},\;x_{3}\right)+p\left({\ddot{x}}_{1}\wedge x_{2},\;x_{3}\right)=\\ & = & g\left({\dot{x}}_{1},\;x_{2},\;x_{3}\right)+g\left({\ddot{x}}_{1},\;x_{2},\;x_{3}\right). \end{array}$$

The mapping *g* is trivariate *s*-map on *L*. □

**Corollary 1.**  *Let L be a quantum logic and x,y be observables on L. If x↔y, then for any observable z and s-map p, there exists the joint distribution $p_{x,y,z}^{3}$ if we know marginal distributions.*

**Corollary 2.**  *Let a QL L consists of n blocks $B_{1},\cdots ,B_{n}$ ($L={⋃}_{i=1}^{n}B_{i}$ and $\left\{0_{L},1_{L}\right\}\subseteq {⋂}_{i=1}^{n}B_{i}$), where $B_{i}$ is a Boolean algebra, i=1,⋯,n. Then any n-variate s-map $p^{n}$ satisfying*

$$p^{n}\left(x_{1},\cdots ,x_{n}\right)=p^{n}\left(\pi \left(x_{1},\cdots ,x_{n}\right)\right)$$

*for any $\left(x_{1},\cdots ,x_{n}\right)\in L^{n}$ and arbitrary permutation $\pi :\;L^{n}\to L^{n}$ of $\left(x_{1},\cdots ,x_{n}\right)$, can be extended to a (n+1)-variate s-map.*

**Proof of Corollary 2.**  With *n* blocks and n+1 elements $x,x_{1},\cdots ,x_{n}$, there must exist at least one $x_{i}$ of $x_{1},\cdots ,x_{n}$ such that $x↔x_{i}$.

Then, similarly to the previous Proposition, we set

$$g\left(x,\;x_{1},\cdots ,x_{n}\right)=p^{n}\left(x\wedge x_{i},\;x_{1},\cdots ,x_{i-1},\;x_{i+1},\cdots ,x_{n}\right).$$

The mapping *g* is (n+1)-variate *s*-map on *L*. □

## 5. Conclusions

We have shown that under the assumption a quantum logic *L* consists of two blocks of Boolean algebras, then any commutative bivariate *s*-map can be extended to a trivariate *s*-map $p^{3}$, which is designed in a specific way following the structure of the map *g* in the proof of Proposition 4. It follows, if x,y are compatible observables on *L*, then for any observable *z* and arbitrary *s*-map *p*, the joint distribution $p_{x,y,z}^{3}$ exists in cases where the marginal distributions are known.

Analogous reasoning as in Proposition 4 can then be extended to n+1 random variables for a quantum logic consisting of at most *n* blocks of Boolean algebras. One can notice that if the number of blocks in a quantum logic *L* is less than the number of variables in the *s*-map, some properties in the extension of *s*-map, like being permutation free, are preserved, i.e., these characterizations carried by *s*-maps and valid within a Boolean algebra can be partially transferred to a larger generalization like a quantum logic.

Unlike the paper [42], in which authors have shown that necessary and sufficient conditions for the existence of trivariate extension $p^{3}$ of a commutative bivariate *s*-map *p* are in compliance with triangle inequalities for the function $d_{p}$ generated by *p*, the results obtained in this paper refer to the maximal number of blocks in a quantum logic *L* guaranteeing the existence of $p^{3}$. If the assumptions of Proposition 4 are fulfilled, there exists the trivariate extension $p^{3}$ of *s*-map *p*, and the compliance with triangle inequalities by $d_{p}$ follows directly.

In addition, Corollary 2 provides another criterion to obtain positive information about the existence of multivariate extension of any permutation-free *s*-map *p*, even in a case when triangle inequalities for the function $d_{p}$ (as the sufficient conditions for extending trivariate *s*-map to quadrivariate *s*-map) fail to hold (see [42]).

The last statement does not indicate that to consider the existence of extension by verifying triangle inequalities is completely wrong. It only claims that this verification is intended for trivariate extension of a bivariate *s*-map rather than for quadrivariate extension of a trivariate *s*-map. The problem of finding the necessary and sufficient conditions for the existence of a quadrivariate extension of a trivariate s-map has not been settled and requires further investigations.

## Figures and Tables

**Table 1:** The values of *p*. For any u,v∈L: p(u,v)=p(v,u).

*p*	*a*	$a^{\prime }$	*b*	$b^{\prime }$	*c*	$c^{\prime }$
*a*	0.1	0	0.1	0	0.1	0
$a^{\prime }$		0.9	0.1	0.8	0.3	0.6
*b*			0.2	0	0	0.2
$b^{\prime }$				0.8	0.4	0.4
*c*					0.4	0
$c^{\prime }$						0.6

## Data Availability

The original contributions presented in this study are included in the article. Further inquiries can be directed to the corresponding author.
